# Neonatal hyperoxia exposure induces aortic biomechanical alterations and cardiac dysfunction in juvenile rats

**DOI:** 10.14814/phy2.14334

**Published:** 2020-01-10

**Authors:** Merline Benny, Diana R. Hernandez, Mayank Sharma, Keyvan Yousefi, Shathiyah Kulandavelu, Sunil Batlahally, Ronald Zambrano, Pingping Chen, Eliana C. Martinez, Augusto F. Schmidt, Lina A. Shehadeh, Roberto I. Vasquez‐Padron, Shu Wu, Omaida C. Velazquez, Karen C. Young

**Affiliations:** ^1^ Department of Pediatrics University of Miami Miller School of Medicine Miami Florida; ^2^ Batchelor Children’s Research Institute University of Miami Miller School of Medicine Miami Florida; ^3^ Department of Surgery University of Miami Miller School of Medicine Miami Florida; ^4^ The Interdisciplinary Stem Cell Institute University of Miami Miller School of Medicine Miami Florida; ^5^ Department of Molecular and Cellular Pharmacology University of Miami Miller School of Medicine Miami Florida; ^6^ Division of Cardiology Department of Medicine University of Miami Miller School of Medicine Miami Florida

**Keywords:** developmental programming, LV dysfunction, neonatal hyperoxia, systemic vascular stiffness

## Abstract

Supplemental oxygen (O_2_) therapy in preterm infants impairs lung development, but the impact of O_2_ on long‐term systemic vascular structure and function has not been well‐explored. The present study tested the hypothesis that neonatal O_2_ therapy induces long‐term structural and functional alterations in the systemic vasculature, resulting in vascular stiffness observed in children and young adults born preterm. Newborn Sprague‐Dawley rats were exposed to normoxia (21% O_2_) or hyperoxia (85% O_2_) for 1 and 3 weeks. A subgroup exposed to 3 weeks hyperoxia was recovered in normoxia for an additional 3 weeks. Aortic stiffness was assessed by pulse wave velocity (PWV) using Doppler ultrasound and pressure myography. Aorta remodeling was assessed by collagen deposition and expression. Left ventricular (LV) function was assessed by echocardiography. We found that neonatal hyperoxia exposure increased vascular stiffness at 3 weeks, which persisted after normoxic recovery at 6 weeks of age. These findings were accompanied by increased PWV, aortic remodeling, and altered LV function as evidenced by decreased ejection fraction, cardiac output, and stroke volume. Importantly, these functional changes were associated with increased collagen deposition in the aorta. Together, these findings demonstrate that neonatal hyperoxia induces early and sustained biomechanical alterations in the systemic vasculature and impairs LV function. Early identification of preterm infants who are at risk of developing systemic vascular dysfunction will be crucial in developing targeted prevention strategies that may improve the long‐term cardiovascular outcomes in this vulnerable population.

## Graphical abstract

Prolonged neonatal oxygen exposure causes persistent aortic structural and biomechanical alterations, which was even more pronounced after recovery in normoxia. This was accompanied by a marked impairment in left ventricular function. These findings have important implications as it suggests that premature babies exposed to oxygen may be predisposed to vascular dysfunction, leading to increased risk of cardiovascular disease in later life. As this affected population begins to age, there will be a critical need to identify the underlying pathophysiological mechanisms for these vascular morbidities in preterm survivors.

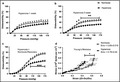

## 1 INTRODUCTION

Early postnatal exposures may have life‐long health and disease implications into adulthood, a concept called developmental programming (Abitbol, DeFreitas, & Strauss, 2016; Barker, 2007; Vaag, Grunnet, Arora, & Brons, 2012). Approximately 12% of all live births in the United States every year are preterm infants (<37 completed weeks) with an estimated annual societal economic burden of over $26 billion (Institute of Medicine Committee on Understanding Premature B, & Assuring Healthy O, 2007). With advances in neonatal care, more than 95% of these preterm infants survive into adulthood. However, this may come at the expense of future adverse health characterized by failure to achieve optimal development and more rapid rates of decline, or “accelerated aging” in organ function (Raju, Buist, Blaisdell, Moxey‐Mims, & Saigal, 2017; Shalev et al., 2014). Recent large epidemiological studies demonstrate a higher risk of hypertension, ischemic heart disease, atherosclerosis, and stroke in adults born preterm (Crump et al., 2019; de Jong, Monuteaux, Elburg, Gillman, & Belfort, 2012; Stritzke, Thomas, Amin, Fusch, & Lodha, 2017; Yzydorczyk et al., 2008). Since preterm infants are exposed to multiple antenatal and neonatal insults, the mechanisms underlying the structural alterations in the systemic vasculature remain unclear.

Most preterm babies are treated with supplemental oxygen (O_2_) therapy, and exposure to a high concentration of O_2_, especially when compared to the relative hypoxic condition of intrauterine life, impairs lung angiogenesis leading to bronchopulmonary dysplasia (BPD) or chronic lung disease of prematurity (Baraldi, Carraro, & Filippone, 2009). Though the effect of O_2_ on long‐term lung development of premature infants has been extensively studied, its effect on the systemic vasculature and heart, which develop in parallel to the lung, is less explored (Abitbol et al., 2016; Nigam, 2013). There is, however, evidence that neonatal hyperoxia exposure increases reactive oxygen species (ROS) production and alters elastin and collagen distribution within the systemic vasculature (Fujinaga et al., 2009; Huyard et al., 2014; Pennathur & Heinecke, 2007). Huyard et al. (2014) exposed rat pups to 80% O_2_ from postnatal days 3 to 10 and demonstrated increased collagen to elastin ratio at 4 weeks. Moreover, Mivelaz et al. (2011) demonstrated increased aortic pulse wave velocity (PWV), a marker of vascular stiffness when the rats were evaluated at 6 and 9 months, suggesting that neonatal hyperoxia plays a crucial role in the pathogenesis of systemic vascular dysfunction in adults born preterm. Despite this, there is limited data on the sequential biomechanical changes in the systemic vasculature induced by neonatal hyperoxia thus hindering the development of effective preventive strategies.

It is now known that prematurity induces persistent alterations in systemic vascular structures (Odri Komazec et al., 2016; Tauzin et al., 2014) which starts early in life (Sehgal, Malikiwi, Paul, Tan, & Menahem, 2016; Tauzin et al., 2006). Vascular stiffness is one of the earliest detectable manifestations of adverse structural and functional changes within the vessel wall and has been shown to precede hypertension (Cavalcante, Lima, Redheuil, & Al‐Mallah, 2011; Takase et al., 2011). Emerging evidence indicates that aortic stiffness is associated with cardiovascular events and impaired function (Cooper & Mitchell, 2019). Large systemic vessels like the aorta, aside from providing a conduit for blood to reach peripheral tissues, play a critical role in providing adequate vascular buffering to each ventricular contraction through arterial‐ventricular coupling (Cavalcante et al., 2011). Therefore, these structural and functional aortic alterations may have significant implications for cardiac function. Recent studies demonstrate left heart dysfunction (Mohlkert et al., 2018) and increased left ventricular mass in adults born preterm (Lewandowski et al., 2013). Whereas it is well‐accepted that neonatal O_2_ alters right ventricular function in young adults born preterm, the effects on long‐term left ventricular function remain unclear.

In the present study, we evaluated vascular stiffness and cardiac function in neonatal rodents exposed to varying periods of O_2_ exposure. We demonstrate that neonatal O_2_ exposure for 1 week shows a trend in decreased aortic distensibility but exposure for 3 weeks causes significant aortic structural and biomechanical alterations, which persists after recovery in normoxia for an additional 3 weeks. These findings were accompanied by a marked impairment in left ventricular function. Our study has important implications for clinical practice as it may provide a better understanding of the sequential changes in vascular stiffness could lead to early interventions and better long‐term outcomes in preterm infants (Sperling, 2004).

## 2 MATERIALS AND METHODS

### 2.1 Ethics statement

The Animal Care and Use Committee at the University Of Miami Miller School Of Medicine approved the protocol. We performed this study in strict accordance with the recommendations in the Guide for the Care and Use of Laboratory Animals of the National Institutes of Health. All surgery was performed under isoflurane anesthesia, and every effort was made to minimize suffering.

### 2.2 Experimental model

Pregnant Sprague‐Dawley rats were obtained from Charles River Laboratories (Wilmington, MA) and were housed with food and water available ad libitum at constant temperature (25°C) under 12:12 light/dark cycle. Rat pups (postnatal day 1) were randomly assigned to normoxia or hyperoxia (85% O_2_) for 1 or 3 weeks. The pups were housed in a plexiglass chamber with continuous O_2_ exposure and monitoring that was briefly interrupted for animal care (<10 min/day). Mothers were rotated every 48 hr between normoxia and hyperoxia chambers to prevent damage to their lungs. Litter size was adjusted to 10–12 pups to control for the effect of litter size on growth and nutrition. We randomly distributed pups from six litters for these studies. Those exposed to hyperoxia for 3 weeks were recovered in normoxia for an additional 3 weeks. Both male and female rats were studied at 1, 3, and 6 weeks, respectively, as they continued to mature.

### 2.3 Ex vivo intrinsic aorta stiffness‐pressure myography

Descending aortas were mounted on steel cannulas (outer diameter‐size range between 400 and 1,200 µm) in a pressure myography system (110P‐DMT‐USA Inc., Danish Myo Technology). An inverted microscope (Leica DMI 6000) equipped with a charge couple device camera was used to visualize the vessels. MyoView II software (Danish MyoTechnology) was used to monitor inside and outside diameters and to record the data. All surrounding fat tissue was removed from the aortas and the aortas were placed on the pressure myograph chamber. After the first cannula was mounted, aortas were cleaned and perfused with HEPES‐buffered Krebs solution containing 2.5 mM CaCl_2_. The vessels were allowed to equilibrate at 45 mmHg and 37°C for 1/2 hr before testing. To examine distensibility, the intraluminal pressure was raised from 5 to 170 mmHg in 10 mmHg increments with a constant temperature at 37°C. The bath solution was changed between samples. The pressure was maintained for 5 min at each step and vessel characterization data were recorded at the end of this time. Distensibility was estimated under relaxed conditions by determining changes in outer diameter size as a function of pressure normalized to the diameter at 5 mmHg.

### 2.4 Calculations of biomechanical parameters

The incremental distensibility of the aorta was studied with a pressure myograph as previously described (Briones, Salaices, & Vila, 2007). Incremental distensibility represents the percentage of change of the arterial diameter for each mmHg change in intraluminal pressure and was calculated according to the formula: Incremental distensibility = ΔDi/(DixΔP) × 100. Di is the internal diameter continuously measured under passive conditions and *p* is the intraluminal pressure.

Arterial stiffness independent of geometry was determined by Young's elastic modulus (*E* = stress/strain) and was calculated according to the method of Briones et al. (2003). A tangential or incremental elastic modulus (*E*_inc_) was calculated by determining the slope of the nonlinear stress‐strain curve (*E*_inc_ = δσ/δε). E_inc_ was obtained by fitting the stress‐strain data from each animal to an exponential curve using the equation: σ = σ_org_exp (βε), where σ_org_ is the stress at the original diameter. Taking derivatives on the above equation, *E*_inc_ = βσ. An increase in β implies an increase in E_inc_, which means an increase in stiffness.

### 2.5 In vivo aorta stiffness‐pulse Wave Doppler

At 6 weeks, we performed high‐resolution ultrasonography measurements of PWV in rat abdominal aorta as previously described (Lee et al., 2016). We used transit time method validated for abdominal aorta using the Vevo2100 imaging system (VisualSonics). PWV was calculated as Δd/Δt, where d is the distance between two points divided by the difference in transit time (t) of the pressure wave arrival at two said points and was measured reproducibly via noninvasive ultrasound micro‐imaging and Doppler ultrasound transit time approach. The transit time was defined as the peak of the ECG R‐wave to the foot of the velocity upstroke with the foot defined as the point at the end of the diastole when the steep rise of the wavefront begins. All measurements were obtained in triplicate; the mean value was used for data analysis. The data were analyzed with VevoLab 1.7.1 software (VisualSonics). The Doppler ultrasound studies were done with the animals sedated with 0.8% isoflurane anesthesia, which is not expected to induce nonphysiological artifacts since it is less than the 1% isoflurane anesthesia level defined as ‘‘baseline level’’ for coronary blood flow with heart rates similar to sleeping rats and documented to not alter aortic impedance (Hartley et al., 2008; Herrera, Decano, Giordano, Moran, & Ruiz‐Opazo, 2014).

### 2.6 Assessment of aortic fibrosis

Distal abdominal aorta segments from 6 week rats were fixed in buffered 4% paraformaldehyde, and serial 5 µm thick paraffin‐embedded sections were prepared. Masson's Trichrome staining was performed to assess aortic collagen deposition, a marker of fibrosis. Sections were evaluated under a light microscope (Leica DMI 6000). Positive staining within the tunica media was assessed by defining the area limited by the tunica intima and the adventitia. Medial fibrosis (% of collagen) was quantified using Image J (National Institutes of Health) and color thresholding methods as previously described (Yzydorczyk et al., 2008). Identical settings and exposure times were used in order to validate comparative analysis between normoxia and hyperoxia rat aorta sections. At least four tissue‐sections per specimen were analyzed by a blinded observer.

### 2.7 Western blot

Total protein was extracted from frozen aortic tissues with a RIPA buffer according to the manufacturer's protocol. The protein concentrations were measured by BCA protein assay using a commercial kit from Pierce Biotechnology Inc. Western blot was performed as previously described (Hummler et al., 2013). Briefly, total proteins (50 μg/sample) were fractionated by SDS‐PAGE on 4%–12% Tris‐glycine precast gradient gels (Invitrogen) and then transferred to nitrocellulose membranes (Amersham). The membranes were incubated overnight at 4°C with a primary antibody for collagen III (Rabbit polyclonal antibody, 1:1,000; Ab 7778, Abcam) and then incubated for 1 hr at room temperature with HRP‐conjugated secondary antibody. Antibody bound protein was detected using ECL chemiluminescence methodology (Amersham). Membranes were then stripped with 0.2 N NaOH and re‐incubated with an anti‐β‐actin antibody (monoclonal antibody, 1:10,000; A5441, Sigma Aldrich). Collagen III expression was analyzed by a Quantity One Imaging Analysis Program (Bio‐Rad) and normalized by β‐actin, a housekeeping protein.

### 2.8 Echocardiography

Echocardiographic evaluation of left ventricular (LV) structure and function was assessed using the Vevo2100 imaging system (VisualSonics). Rats were lightly anesthetized with 1% isoflurane to achieve a heart rate (HR) of 300–400 beats/min and placed on a heated stage. Three measurements at different cardiac cycles were assessed and used for analysis. M‐mode images were obtained in the parasternal short axis view at the level of the papillary muscles to assess LV end systolic diameter (LVES_d_) and LV end diastolic diameter (LVED_d_). Stroke volume (SV) was determined using Doppler flow Velocity‐Time Integral (VTI) at the LV outflow tract (LVOT) and the aortic diameter (Ao), (LVOT^2^ × 0.785 × Ao VTI). Cardiac output was calculated from stroke volume multiplied by heart rate (SV × HR). Systolic function was assessed using M‐mode calculations of fractional shortening (FS = LVED_d_ − LVES_d_/LVED_d_). LV end diastolic volume (LVEDV) was calculated from LVED_d_(7/(2.4 + LVED_d_) × LVED_d_^3^). LV end systolic volume (LVESV) was calculated from LVES_d_(7/(2.4 + LVES_d_) × LVES_d_^3^) and ejection fraction (EF = (LVEDV − LVESV)/LVEDV × 100) as previously described (Velten et al., 2011). Rats were allowed to recover from anesthesia after the determination of all echocardiographic endpoints.

### 2.9 Statistics

Data are expressed as mean ± *SEM*. Statistical significance was evaluated by unpaired Student's *t*‐test. One‐way ANOVA with Bonferroni's correction for multiple comparisons was used to evaluate differences among groups for pressure myography. A *p* < .05 was considered significant. All analyses were performed using commercially available statistical software packages (GraphPad Prism version 8.0 for Windows, GraphPad Software).

## 3 RESULTS

### 3.1 Effect of neonatal hyperoxia on body weight and tibial length

Male and female neonatal rats were exposed to normoxia or hyperoxia (85% O_2_) for 1 week and 3 weeks. A subgroup exposed to 3 weeks of hyperoxia was recovered in normoxia for an additional 3 weeks. Bodyweight tended to be lower in rats exposed to 1 week or 3 weeks of hyperoxia but this was not statistically significant. Moreover, while the tibial length tended to be lower in the hyperoxia exposed group at 6 weeks, this was not statistically significant (Table 1). While there was no difference in mortality following 1 week of hyperoxia exposure, there was a 30% increased mortality in the pups exposed to hyperoxia for 3 weeks when compared to the control groups.

**Table 1:** Age, body weight, gender, tibial length in normoxia and hyperoxia exposed animals

Age	1 week			3 week			6 week		
Exposure	Normoxia	Hyperoxia	*p* value	Normoxia	Hyperoxia	*p* value	Normoxia	Hyperoxia	*p* value
Weight (g) Females	14.2 ± 0.7	13.0 ± 0.5	.2	62.0 ± 2.2	55.3 ± 2.6	.06	183.9 ± 5	179.8 ± 7	.7
Weight (g) Males	14.3 ± 0.1	13.3 ± 0.9	.18	65.0 ± 3.1	58.1 ± 4.4	.14	244.5 ± 5	207.0 ± 14	.06
Tibial length (cm)	1.2 ± 0.1	1.0 ± 0.1	.1	2.0 ± 0.1	1.9 ± 0.1	.1	3.2 ± 0.1	3.1 ± 0.1	.5

Note

*n* = 5–8/group, Data are mean ± *SEM*.

### 3.2 Neonatal hyperoxia decreases aortic distensibility and elasticity in juvenile rats

**Figure 1:**
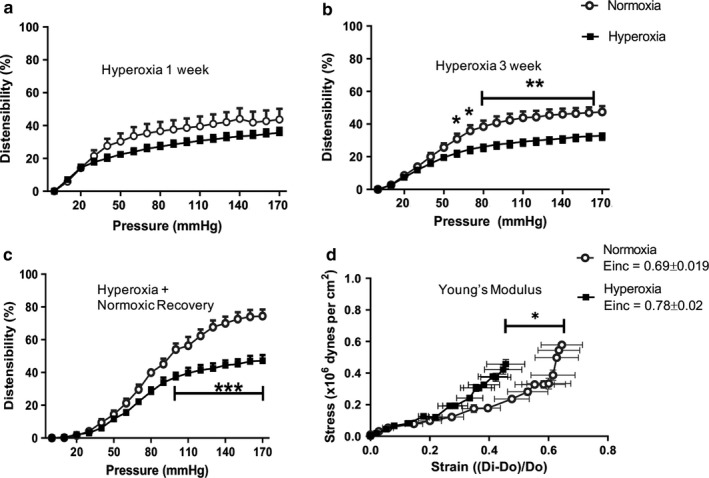
Neonatal hyperoxia alters aortic biomechanics and increases vascular stiffness in 6 week rats. Pressure myography assessment of distensibility of the abdominal aorta at 1 week (a), 3 (b), and 6 weeks (c). Hyperoxia has a trend to decrease distensibility at 1 week and decreases vascular distensibility at 3 and 6 weeks. Pressure myography assessment of stress‐strain curve in abdominal aorta shows increased vascular stiffness in rats exposed to hyperoxia for 3 weeks and recovered in normoxia for 3 weeks (Einc = 0.78 ± 0.02) compared to control group(Einc = 0.69 ± 0.01) at 6 weeks (d). *n* = 5/group, data are mean ± *SD*; one‐way ANOVA with Bonferroni's correction for multiple comparisons were used to evaluate differences among groups. *p < .01; **p < .01 and ***p < .001; normoxia versus hyperoxia

Accumulating evidence suggests that pressure myography is an invaluable method to study the biomechanical properties of the vasculature (Shahid & Buys, 2013). Exposure of neonatal rats to 1 week of hyperoxia showed a trend to decreased aortic distensibility (Figure 1a). However, following 3 weeks of neonatal hyperoxia exposure, the incremental distensibility was significantly decreased in the hyperoxia exposed group compared to the normoxia group (Figure 1b). Moreover, even after recovery in normoxia for three additional weeks, there was a persistent reduction in incremental distensibility in the neonatal hyperoxia exposed 6 weeks old rats (Figure 1c). In addition, aortas from the hyperoxic group exposed to O_2_ for 3 weeks and recovered in normoxia for 3 weeks showed decreased elasticity as evidenced by β (0.69 ± 0.02 vs. 0.78 ± 0.02) and a leftward shift of the stress‐strain relationship when compared to the control group; normoxia versus hyperoxia, *p* < .05, *n* = 5/group, (Figure 1d). This suggests that prolonged neonatal hyperoxia during the critical period of vascular development progressively increases aortic stiffness in growing rats.

### 3.3 Neonatal hyperoxia increases aortic stiffness in juvenile rats

**Figure 2:**
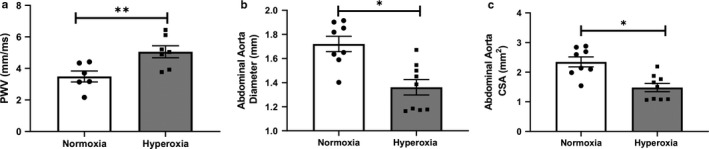
Neonatal hyperoxia exposure increases aortic stiffness and morphology of aorta in 6 week rats. Doppler ultrasound assessment of aortic stiffness shows increased pulse wave velocity (a), decreased diameter (b), and cross‐sectional area (CSA) (c) in the abdominal aorta of adult rats exposed to hyperoxia compared to normoxia. *n* = 6–10/group, data are mean ± *SEM*, Student's unpaired *t*‐test. *p < .05, **p = .01; normoxia versus hyperoxia

Pulse wave velocity (PWV) is an indirect measure of the biophysical properties of a vessel's arterial stiffness, which is a well‐recognized independent risk factor for cardiovascular events such as primary coronary events, stroke, and mortality (Blacher & Safar, 2005). Rats exposed to neonatal hyperoxia for 3 weeks and recovered in normoxia for an additional 3 weeks had a significant increase in PWV (3.5 ± 0.3 vs. 5.1 ± 0.4 m/sec; normoxia vs. hyperoxia, *p* = .01, *n* = 6–7/group, Figure 2a). This was accompanied by a significant decrease in aortic diameter (1.7 ± 0.1 vs. 1.4 ± 0.1 mm; normoxia vs. hyperoxia, *p* < .05, *n* = 8–9/group, Figure 2b) and cross‐sectional area (2.3 ± 0.2 vs. 1.5 ± 0.1 mm^2^; normoxia vs. hyperoxia, *p* < .05, *n* = 8–9/group, Figure 2c respectively). These findings indicate that neonatal hyperoxia contributes to mechanical and structural changes in the aorta of growing rats.

### 3.4 Neonatal hyperoxia induces aortic fibrosis in juvenile rats

**Figure 3:**
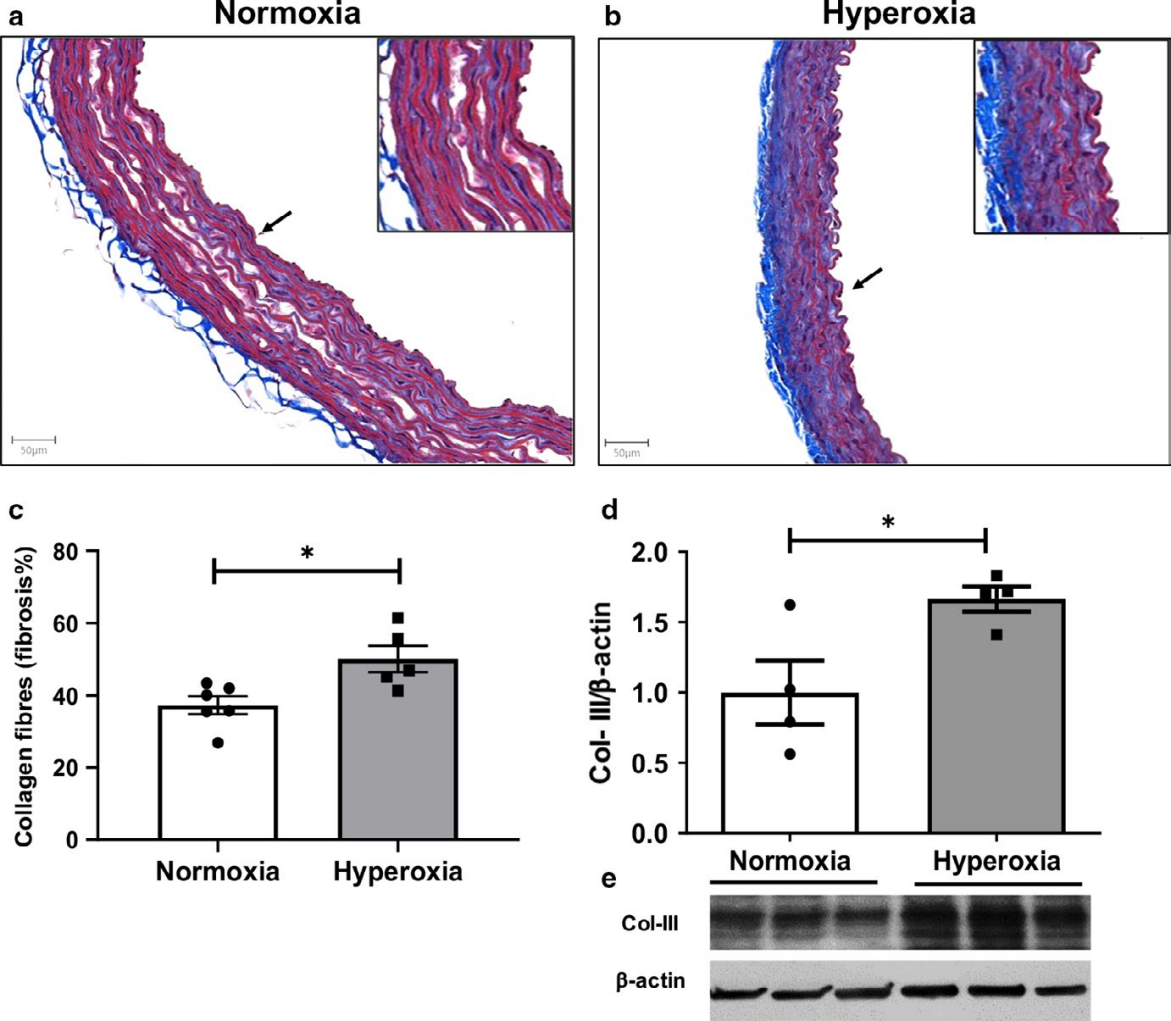
Neonatal hyperoxia exposure increases collagen expression in aortic media of 6 week rats. Representative imgaes of the layers of aorta stained with Masson trichrome showing collagen deposition (blue) in the aortic media of normoxia (a) and hyperoxia (b) exposed rats. Representative images (×20 magnification) of aorta sections are shown; scale bar 50 µm. An inset at 2 × focal enlargement of the areas indicated by the arrows is also given. On quantification using image J, the percent media of the aorta that was stained with collagen was higher in the hyperoxic aorta (c). Representative Western blot analysis of collagen III (Col III) expression (d). Hyperoxia increased Col III expression in the aorta protein extracts (e). *n* = 4–6/group; data are mean ± *SEM*, Student's unpaired *t*‐test. *p < .05; normoxia versus hyperoxia

Compared to normoxic rats, Masson's trichrome stain revealed a significant increase in aortic fibrosis in 6 weeks old rats exposed to neonatal hyperoxia (Figure 3a and b). Densitometric quantitative analysis using Image J software demonstrated a significant increase in collagen fibers in the aortas of hyperoxia exposed 6 weeks old rats (Figure 3c). Collagen III is one of the major forms of collagen in the aorta. Western blot confirmed a 1.6‐fold increase in collagen III expression in these aortas (Figure 3d and e). Taken together these findings suggest that enhanced collagen deposition plays a vital role in vascular remodeling in hyperoxia exposed aorta.

### 3.5 Neonatal hyperoxia induces LV dysfunction in juvenile rats

**Figure 4:**
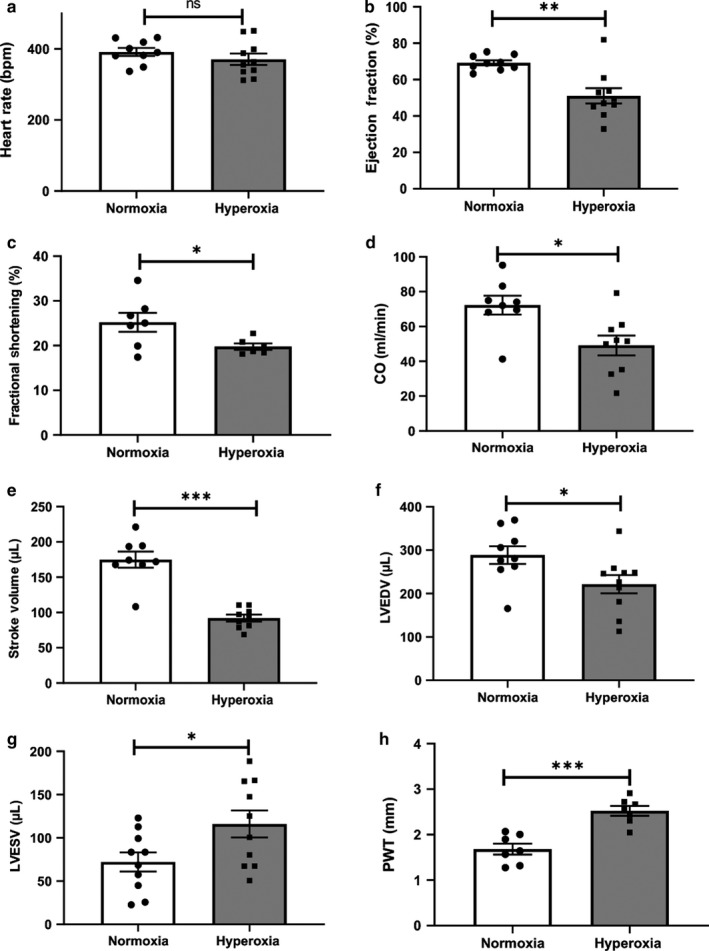
Neonatal hyperoxia exposure decreases LV function in 6 week rats. Functional and morphological parameters of the left ventricle achieved by echocardiography in neonatal normoxia or hyperoxia exposed rats at 6 weeks of age. (a–h). *n* = 7–10/group, data are mean ± *SEM*, Student's unpaired *t*‐test. *p < .05, **p < .01, ***p < .001

Echocardiographic parameters of LV structure and function were assessed at 6 weeks (Figure 4a–h). There was no difference in heart rate between the groups (Figure 4a). There was a significant decrease in LV ejection fraction (Figure 4b), fractional shortening (Figure 4c), cardiac output (Figure 4d), stroke volume (Figure 4e), and LV end diastolic volume (Figure 4f). These changes in hyperoxic rats were accompanied by significantly increased LV end systolic volume (Figure 4g) and posterior wall thickness (Figure 4h). These data suggest that neonatal hyperoxia can cause cardiac dysfunction.

## 4 DISCUSSION

Vascular dysfunction is a well‐recognized consequence of preterm birth with effects into adulthood. Adults born preterm have an increased risk of vascular stiffness, hypertension, and cardiovascular morbidities (Crump et al., 2019). In our current study, we demonstrate that neonatal hyperoxia causes a persistent increase in aortic stiffness, alters its biomechanical properties and induces LV dysfunction in juvenile rats. To the best of our knowledge, the current study is the first to evaluate the sequential changes in vascular stiffness following neonatal O_2_ exposure and the first to demonstrate vascular stiffness of the aorta using pressure myography in the hyperoxia exposed rat model. These findings have important implications as it suggests that supplemental O_2_ may later induce cardiovascular dysfunction in preterm survivors.

The neonatal hyperoxia rat model is a well‐recognized model of preterm birth‐related conditions, such as BPD, retinopathy of prematurity, and brain injury (Alapati et al., 2014; Kim et al., 2019; Ozgurtas et al., 2016; Reiter et al., 2017). It recapitulates the condition of prematurity where antioxidant defense is deficient and supplemental O_2_ renders them vulnerable to oxidative damage (Saugstad, 2005; Thibeault, 2000). We have broadened this model to study the early and long‐term impact of neonatal O_2_ exposure on the systemic vasculature and cardiac function. Aortic pressure myography revealed that rats exposed to 1 week O_2_ had a trend to decreased aortic distensibility, however, 3 weeks O_2_ exposure decreased aortic distensibility which persisted even after recovery in normoxia for an additional 3 weeks. We also confirmed that it is feasible to assess the aortic distensibility by pressure myography and validated it with PWV.

Vascular elasticity is a crucial determinant of blood flow dynamics in the circulatory system (Shadwick, 1999). Systemic arteries must be distensible to provide capacitance and pulse‐smoothing in the circulation, but must also be stable to inflation over a range of blood pressure. These mechanical requirements are met by strain‐dependent increases in the elastic modulus of the vascular wall, manifested by the J‐shaped stress‐strain curve (Shadwick, 1999). The Young's module of elasticity, a marker of arterial stiffness, is a plot of the applied stress (i.e. force/cross‐sectional area) versus the resulting strain (i.e. Δ dimension/initial dimension) on the vasculature (Briones et al., 2003). A higher Young's modulus implies a stiffer vessel wall. Using pressure myography, we found that in neonatal hyperoxia exposed rats, the stress‐strain curve was shifted to the left with a higher Young Modulus, indicating stiffness in the hyperoxia exposed aortas. These findings are suggestive of early aortic stiffness following hyperoxia exposure and are consistent with the results by Sehgal et al that demonstrated increased aortic stiffness as early as 36 weeks postmenstrual age in preterm infants with prolonged O_2_ therapy (Sehgal et al., 2016).

We also corroborated our ex vivo pressure myography findings with in vivo PWV, an indirect measure of arterial stiffness (Mitchell et al., 2010). We demonstrated a significant increase in aortic PWV in 6 week old rats exposed to neonatal hyperoxia. Our findings are consistent with those of Mivelaz et al. (2011) who demonstrated increased PWV in 6 and 9 months old rats exposed to 80% O_2_ from postnatal day 3–10. However, 6–9 month rats correlate with 18–25 human years (Sengupta, 2013). Our present study is the first to assess the effect of neonatal hyperoxia on aortic stiffness using PWV in young rats corresponding to periadolescent children.

Arterial wall stiffness is regulated by factors inherent to the structural composition of the wall, including collagen fibers, elastin fibers, and smooth muscle cells. Fibrosis is characterized by excessive deposition of extracellular matrix and cross‐linking proteins, in particular, collagen leading to vascular stiffening. In our study, we found increased collagen III expression in the media of the aorta obtained from juvenile rats exposed to neonatal hyperoxia. This is in agreement with Huyard et al. (2014) who demonstrated increased collagen in the aortic media of 4 week old rats exposed to 80% O_2_ from postnatal day 3–10.

In addition to the increased collagen deposition in the arterial wall of the hyperoxia exposed group, we also demonstrated a significant decrease in the aortic diameter and cross‐sectional area. These findings are potentially mediated by exaggerated production of superoxide (Yzydorczyk et al., 2008) and are consistent with (Kumar et al., 2018) who demonstrated a significantly smaller diameter in 9‐month‐old mice exposed to neonatal hyperoxia.

Given the intricate relationship between the systemic vasculature and cardiac afterload, we also investigated the long‐term effects of O_2_ on cardiac function. In our study, young rats exposed to neonatal hyperoxia had a pronounced reduction in LV function. Increased LV posterior wall thickness could be attributed to increased aortic stiffness, which may increase afterload resulting in greater LV work, creating a vicious cycle leading to further increases in LV wall thickness and decreased LV filling. We not only demonstrated decreased LV end diastolic volume but also a pronounced reduction in LV ejection fraction and fractional shortening. These findings are consistent with other investigators who have shown impaired cardiac function and fibrosis in adult rodents exposed to neonatal hyperoxia (Bertagnolli et al., 2014). It should however be noted that the LV dysfunction in our study may be secondary to pulmonary hypertension (Abman, Grenolds, & Mourani, 2016), as other studies in our lab have demonstrated significantly increased RV systolic pressure in young rats exposed to neonatal hyperoxia (Bryan et al., 2019). In our present study, we did not evaluate the degree of pulmonary hypertension but we postulate that our current findings of impaired LV function may be secondary to the effects of neonatal O_2_ exposure on the systemic vasculature or potentially secondary to pulmonary hypertension.

The strengths of the current study include the multiple methods deployed to demonstrate vascular function, involving both in vivo and ex vivo detailed longitudinal biomechanical assessments and the clinically relevant nature of the study. Our study, however, has certain limitations. The phenotype observed in our model is severe and may amplify the disease evidenced in premature infants. We however chose 1 week of oxygen exposure in neonatal rodents as it represents an equivalent stage of human development from 24–38 weeks gestational age and 21 days of oxygen exposure as this is equivalent to 18–24 human months and would represent an infant population needing prolonged oxygen support (Nardiello, Mižíková, & Morty, 2017). In addition, although our study demonstrated that hyperoxia plays a role in the pathogenesis of vascular stiffness, there are several other factors including small for gestation, poor nutrition, preeclampsia, gestational diabetes, which may also contribute to it. Future studies to investigate some of these risk factors with or without combined O_2_ exposure, their mechanistic role in vascular stiffness, its relationship with onset of hypertension, and cardiac remodeling will be important.

In conclusion, our study has elaborated on our knowledge of the known aortic phenotype of vascular stiffness in survivors of preterm birth who were exposed to O_2_ therapy in neonatal period. We identified the sequential alterations in aortic vascular stiffness induced by O_2_ in a rat model, as a basis to understand the structural and biomechanical changes in the systemic vasculature that preterm survivors are known to exhibit. Though the impact of adverse perinatal environmental exposures may not be evident until additional risk factors are introduced during childhood and adulthood, understanding the early changes and the long‐term consequences could provide novel approaches to the development of interventional strategies.

## CONFLICT OF INTEREST

None declared.
